# Self-assembly and Hydrogelation Properties of Peptides Derived from Peptic Cleavage of Aggregation-prone Regions of Ovalbumin

**DOI:** 10.3390/gels8100641

**Published:** 2022-10-09

**Authors:** Raliat O. Abioye, Caleb Acquah, Pei Chun Queenie Hsu, Nico Hüttmann, Xiaohong Sun, Chibuike C. Udenigwe

**Affiliations:** 1 Department of Chemistry and Biomolecular Sciences, Faculty of Science, University of Ottawa, Ottawa, ON K1N 6N5, Canada; 2 School of Nutrition Sciences, Faculty of Health Sciences, University of Ottawa, Ottawa, ON K1H 8M5, Canada; 3 Department of Bioengineering, Faculty of Life Sciences, Rhine-Waal University of Applied Sciences, Marie-Curie-Straße 1, 47533 Kleve, Germany; 4 Department of Plant, Food, and Environmental Sciences, Faculty of Agriculture, Dalhousie University, Truro, NS B2N 5E3, Canada

**Keywords:** peptide self-assembly, egg white hydrolysates, ovalbumin, peptide hydrogels, rheology, biomechanical properties

## Abstract

Egg white protein hydrolysate generated with pepsin was investigated for the presence of peptides with self-assembly and hydrogelation properties. Incubation of the hydrolysates for 16 h resulted in aggregates with significantly (*p* < 0.05) lower free amino nitrogen and sulfhydryl contents, and higher particle diameter and surface hydrophobicity compared to the hydrolysates. LC-MS/MS analysis of the aggregates resulted in identification of 429 ovalbumin-derived peptides, among which the top-six aggregation-prone peptides IFYCPIAIM, NIFYCPIAIM, VLVNAIVFKGL, YCPIAIMSA, MMYQIGLF, and VYSFSLASRL were predicted using AGGRESCAN by analysis of the aggregation “Hot Spots”. NIFYCPIAIM had the highest thioflavin T fluorescence intensity, particle diameter (5611.3 nm), and polydispersity index (1.0) after 24 h, suggesting the formation of β-sheet structures with heterogeneous particle size distribution. Transmission electron microscopy of MMYQIGLF, and VYSFSLASRL demonstrated the most favorable peptide self-assembly, based on the formation of densely packed, intertwined fibrils. Rheological studies confirmed the viscoelastic and mechanical properties of the hydrogels, with IFYCPIAIM, NIFYCPIAIM, VLVNAIVFKGL, and VYSFSLASRL forming elastic solid hydrogels (tan δ < 1), while YCPIAIMSA and MMYQIGLF formed viscous liquid-like hydrogels (tan δ > 1). The results provide valuable insight into the influence of peptide sequence on hydrogelation and self-assembly progression, and prospects of food peptides in biomaterial applications.

## 1. Introduction

Egg white is a common ingredient used in a myriad of food products, including mayonnaise, baked goods, meringues, souffles, custards, and confectioneries [1]. This is due to its low cost and attractive functional properties, such as gelling, emulsifying, and foaming capacities [2,3]. Egg white is highly digestible and nutritious, and represents about 56% of proteins (ovalbumin, conalbumin, ovomucoid, globulins, ovomucin, lysozyme, and flavoprotein) in eggs [4,5]. Protein cross linking facilitated via disulfide bond formation is the main factor that drives the gelling of egg white. After denaturation, the sulfhydryl groups of ovalbumin and ovotransferrin become exposed to the environment, enhancing sulfhydryl reactions to form disulfide bonds, thus promoting the formation of protein gels [6]. Additionally, the high viscosity of ovomucin contributes to the gelling properties of egg white and plays an important role in gel formation. Other proteins that influence gelling of egg white include ovomucoid and lysozyme [6]. The process of formation for most gels follows the general steps of conformational change or moderate denaturation of the protein molecule followed by self-association and subsequent gelation. Minor denaturation or conformational change is required to facilitate the exposure of the aggregation-prone regions of the protein/peptide. Environmental conditions are generally altered to trigger this effect. The most common, especially in the case of egg white, is heat-induced gelling; others include changes in pH or salt concentration, or the addition of monovalent and/or divalent cations [6,7]. Formation of a stable gel is driven by covalent and non-covalent interactions, and results in a change of the surface properties, number of free sulfhydryl groups, and solubility of the denatured protein molecules [6].

Food-derived bioactive peptides have gained significant interest in the past two decades in health and biomaterial applications. Bioactive peptides from egg white proteins have been demonstrated to exert important physiological functions, such as antioxidative, anticancer, antimicrobial, and antihypertensive activities, in the human body [1,8,9]. Owing to their unique physicochemical properties, peptides are increasingly used in the fabrication of hydrogels in the design of new food products and oral delivery systems, and in regenerative medicine [10]. Hydrogels are three-dimensional cross-linked polymers with the ability to swell in water or gastrointestinal biological fluids and retain large quantities of the fluid within their structure [7,11]. Peptide hydrogels have several potential applications, including as controlled-release drug delivery systems. An example is the pH-specific release of anticancer drugs near the tumor site using peptide hydrogels [12,13]. Alternatively, delayed release can be achieved by adjusting the peptide concentration to enhance networking of the hydrogel, thus prolonging resistance to proteolytic degradation. For example, mixing of antidiabetic hempseed hydrolysates with the hydrogel forming RADA16 peptide at a 1:3 ratio (hydrolysate:RADA16) ensured the biostability of the anti-dipeptidyl peptidase-IV activity of the hydrolysates [14]. Owing to their ultra-natural state, food peptides are generally safe and have potential bioactivities, making them an excellent starting material for producing functionalized peptide hydrogels [15]. Furthermore, they are easily mass-producible via solid phase peptide synthesis and structurally modifiable to better suit their intended applications [7,15]. Peptides are also easily tunable by adjusting the physical environment such as pH, temperature, and ionic strength, amongst other external stimuli, providing avenues in more targeted and environment-sensitive applications [15]. However, biostability is a common challenge given the susceptibility of peptides to proteolytic degradation [11]. Thus, some peptides are structurally modified to enhance their biostability to support the formation of self-supporting nanostructures including fibrils, nanotubes, nanofibers, and cross-β structures, whilst maintaining applications in tissue engineering and drug delivery systems [12,16,17].

Ovalbumin is the most abundant egg white protein (EWP), accounting for more than 50% of the total proteins [18]. Several studies have demonstrated the use of ovalbumin as nanogels and nanocarriers when combined with surfactants, metal ions, and chitosan [19,20,21]. Notably, the ovalbumin epitope, SIINFEKL, which has been extensively used for immune stimulation, was reported to self-assemble to form a stable hydrogel [22]. These studies indicate the potential of ovalbumin for the discovery of other hydrogelling peptides. Given its predominance in egg white and ability to form a highly ordered molten globule, ovalbumin was selected as the most promising parent EWP for the discovery of peptide hydrogels. Therefore, the objectives of this study were to identify ovalbumin peptides with hydrogel forming properties using enzymatic hydrolysis and LC-MS/MS analysis, predict the top aggregation-prone peptides by in silico analysis, synthesize the peptides, and characterize the peptide hydrogels using thioflavin T kinetics, dynamic light scattering, transmission electron microscopy, and biomechanical characterization.

## 2. Results and Discussion

### 2.1. Physicochemical and Surface Properties of EWP Hydrolysate and Aggregate Samples

**Figure 1:**
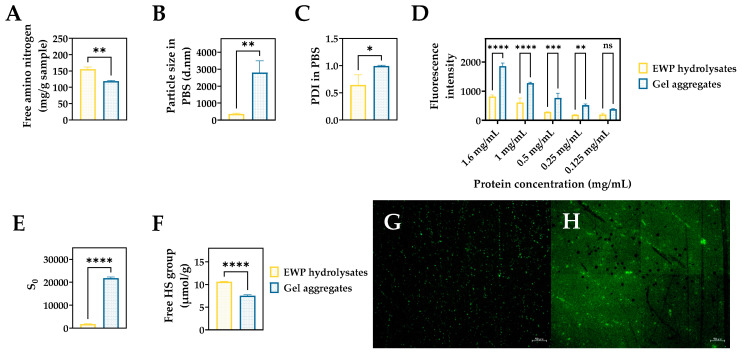
(**A**) Free amino nitrogen content, (**B**) particle diameter (**C**) polydispersity index (PDI), (**D**) ThT fluorescence intensity at different sample concentrations, (**E**) surface hydrophobicity (S0), and (**F**) free sulfhydryl group content of EWP hydrolysate and gel aggregate samples. ThT fluorescence images of (**G**) EWP hydrolysates and (**H**) gel aggregates at 1.6 mg/mL. ns = not significant (*p* ≥ 0.05), * = significant (0.01 < *p* < 0.05), ** = very significant (0.001 < *p* < 0.01), *** = extremely significant (0.0001 < *p* < 0.001), **** = extremely significant (*p* < 0.0001). Scale bars represent 50 µm.

To observe the potential of EWP hydrolysates as a source of peptide hydrogels, the extent of aggregation after 16 h of incubation was evaluated by analysis of free amino nitrogen, particle diameter, polydispersity index, free sulfhydryl group content, and ThT fluorescence intensity. Compared to EWP hydrolysates, the gel aggregate sample showed significantly (*p* < 0.05) lower free amino nitrogen content, and higher particle size and PDI values (Figure 1A–C), suggesting the formation of aggregates. These results were confirmed with ThT fluorescence assay, which also showed a concentration-dependent increase in fluorescence intensity of the aggregates relative to EWP hydrolysates (Figure 1D). An increased ThT fluorescence is indicative of the presence of β-sheet structures, resembling a cross-β sheet arrangement commonly present in fibrils [23]. Furthermore, there was a significant increase in surface hydrophobicity of the aggregate sample compared to EWP hydrolysates (Figure 1E). Surface hydrophobicity is expected to decrease with hydrogelation, a common feature attributed to the involvement of hydrophobic cavities in driving subsequent aggregation [24]. However, it is also possible that the significant increase in the surface hydrophobicity of the gel aggregates is a result of the increased exposure of hydrophobic regions due to molecular rearrangement of the interacting peptides. The significantly higher ThT fluorescence intensity of the aggregate sample confirmed the increased presence of aggregates with increasing concentration compared to the hydrolysates (Figure 1D). Fluorescence microscopy images (Figure 1G-H) showed the presence of larger sized puncta in the gel aggregate sample. This suggests the formation of larger nuclei, which can serve as a template for further growth of fibrillar structures during the peptide aggregation process [11]. A similar increase in ThT fluorescence was observed with self-assembling RADA16 peptide, likely also due to conformational changes within the peptide that promoted strong amyloidogenic-like features commonly seen in β-sheet structures [25]. Compared to the EWP hydrolysates, significantly decreased free sulfhydryl group content was observed in the gel aggregate sample (Figure 1F). This finding suggests that disulfide bonds could be involved in the formation of the aggregates [26]. Intramolecular disulfide bond formation provides stability to the hydrogels through cross-linking [27]. Conversely, hydrophobic interactions can enhance particle formation resulting in accelerated aggregation and hydrogelation, a mechanism suggested as the driving force for many food-derived hydrogels from milk hydrolysates and plant proteins [11], and likely the EWP hydrolysates in this study.

### 2.2. Peptidomic Profile of EWP Hydrolysate and Aggregate Samples

**Figure 2:**
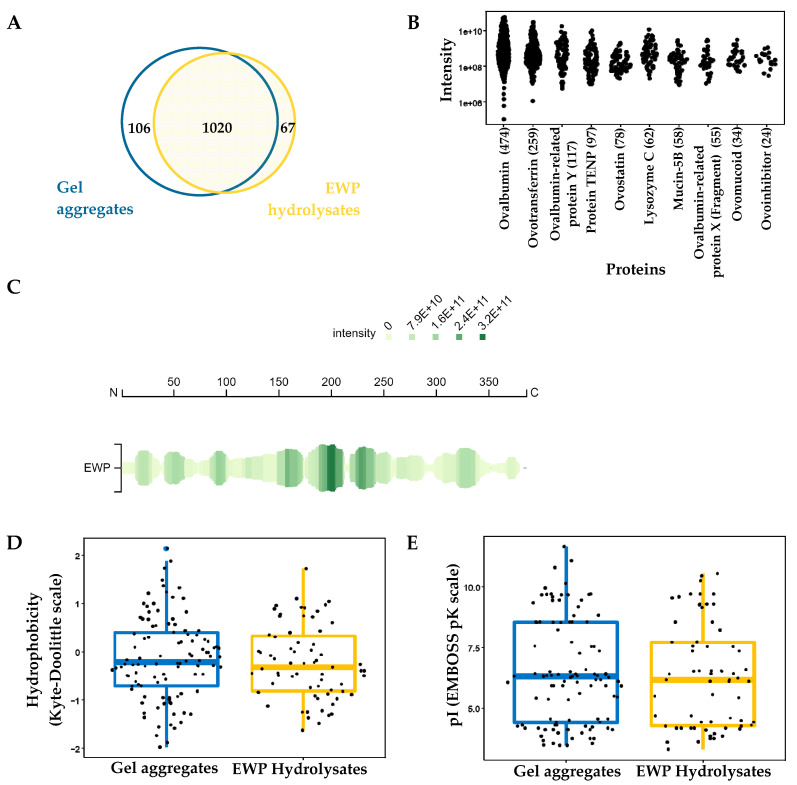
(**A**) Number of peptides identified in both samples and (**B**) the protein origin of the peptides. (**C**) Coverage of the ovalbumin peptides. (**D**) Distribution of hydrophobicity scores and (**E**) the isoelectric points (pI) of peptides from the gel aggregate and EWP hydrolysate samples.

LC-MS/MS of EWP hydrolysates produced with pepsin and aggregates resulted in the identification of 1193 unique peptides, with 85% overlap between the samples. Of the total, 106 peptides were identified exclusively in the gel aggregate sample while 67 peptides were identified in the EWP hydrolysate sample (Figure 2A). Some peptides identified exclusively in the aggregate sample contain miscleavages occurring mostly at W/E, Q/E, and E/D, such as KGLWEKAFKDE and DTREMPFSMTKEESKPVQM. A higher number of miscleavages was found in peptides identified in EWP hydrolysates, especially adjacent to lysine (G/L, L/L, L/V, L/T, L/K, and I/L), aspartate (D/C, D/G, V/D, and D/E), arginine (N/V, F/N, and N/I), and alanine residues (T/A, A/I, and Q/I). Miscleavage, a common feature of enzymatic hydrolysis of proteins, is dependent on the nature of the enzyme, protein substrate, reaction conditions, and amino acid residues at the miscleavage site. Approximately 70% of the identified peptides originated from ovalbumin (and related proteins) and ovotransferrin, which accounted for 49.7% and 21.5% of the peptides, respectively (Figure 2B). From ovalbumin in particular, the coverage of the peptide dataset was 98%, which was almost complete, since the signal peptide sequence was not part of the dataset (Figure 2C). Other parent proteins of identified peptides included TENP protein (7.7%), ovostatin (6.2%), lysozyme C (4.9%), mucin 5B (4.6%), ovomucoid (2.7%), and ovoinhibitor (1.9%) (Figure 2B).

Peptide length distribution of the EWP hydrolysates and aggregates are comparable, with the majority ranging from 9 to 18 residues. Similar distributions of residue types were observed in both samples, with the most frequent types consisting of small, non-polar and polar residues. Polar and non-polar amino acids participate in hydrogelation, as alternating hydrophobic and hydrophilic amino acid arrangement favors the formation of β-sheets [11]. Specifically, anionic and cationic amino acids of the hydrophilic face of β-sheets enable electrostatically driven self-assembly of peptides [11]. A large fraction of both samples had a hydrophobicity score below zero, indicating that the identified peptides were mostly hydrophilic [28], except for the smaller peak appearing in the hydrolysate at a higher hydrophobicity score of approximately 1. This peak resulted from the higher number of hydrophobic residues in the EWP hydrolysate sample (Figure 2D). However, the higher molecular hydrophobicity could not explain the significantly lower surface hydrophobicity of the hydrolysate compared to the aggregate sample (Figure 1E). Additionally, amino acid composition was mostly similar in the hydrolysate and aggregate samples, except for the slightly higher proportion of Ala, Phe, Pro, and Val in EWP hydrolysate, and Arg, Asp, Cys, Gly, Ile, and Leu in the aggregates. These slight differences resulted in the highest number of peptides with an isoelectric point of 6.0 for the aggregate sample compared to ~4.5 for the hydrolysate (Figure 2E).

### 2.3. Predicted Aggregation Propensity of Identified Peptides

As the major component of EWP with self-assembly property and parent protein of one-third of the identified peptides, ovalbumin was selected for further analysis. The 429 ovalbumin-derived peptides showed a wide range of aggregation propensity as predicted using AGGRESCAN (Table S1). The normalized a^4^v sequence sum for 100 residues (Na^4^vSS) is a parameter used for the prediction of protein aggregation propensity [29]. The top-six ranked peptides with distinct sequences (Table 1) were selected for further investigation. Aggregation propensity is based on short sequences of 5–11 residues, their sequence composition, the individual amino acid aggregation propensities, and the position of specific amino acids relative to one another [29]. For example, proline is an aggregation breaker and a region with five or more consecutive residues in a peptide sequence without proline is termed an aggregation “Hot Spot”. Likewise, NIFYCPIAIM and YCPIAIMSA were also selected to examine the effect of “Hot Spots” on peptide self-assembly as they have a common region (YCPIAIM) with the highest-ranking IFYCPIAIM, and their sequence differences resulted in a substantial increase in the total hot-spot area and a decrease in the Na^4^vSS values, respectively. Interestingly, the Na^4^vSS values correlated positively with the hydrophobicity score of the peptides (Table 1). All six peptides were identified in both the EWP hydrolysate and aggregate samples, except YCPIAIMSA, which was identified in only the latter.

### 2.4. Self-Assembly Properties of the Aggregation-Prone Peptides

The six aggregation prone ovalbumin peptides were synthesized and their critical aggregation concentration (CAC), the concentration threshold where aggregation occurs spontaneously, was evaluated using ThT, which has the propensity to bind β-sheet rich regions of fibrils formed after peptide self-assembly [33]. Thus, an increase in fluorescence intensity indicates a higher β-sheet content. The CAC of the ovalbumin-derived peptides, from the lowest to highest values, were 5.14 ± 0.94 µM (VLVNAIVFKGL), 5.35 ± 0.11 µM (VYSFSLASRL), 5.79 ± 0.07 µM (NIFYCPIAIM), 5.95 ± 0.47 µM (MMYQIGLF), 6.22 ± 0.03 µM (YCPIAIMSA), and 9.52 ± 0.73 µM (IFYCPIAIM) (Figure S1). Although selected based on the propensity of the peptide sequences to aggregate and on amyloidogenic properties of individual amino acid sequence [29,34], there was no apparent correlation between the CAC and Na^4^vSS score or total hot-spot area. Thus, while AGGRESCAN is an important tool for the prediction of the propensity of peptides to aggregate, it may not accurately predict the extent of aggregation; hence, a higher score does not necessarily result in a lower CAC or faster aggregation progression. 

**Figure 3:**
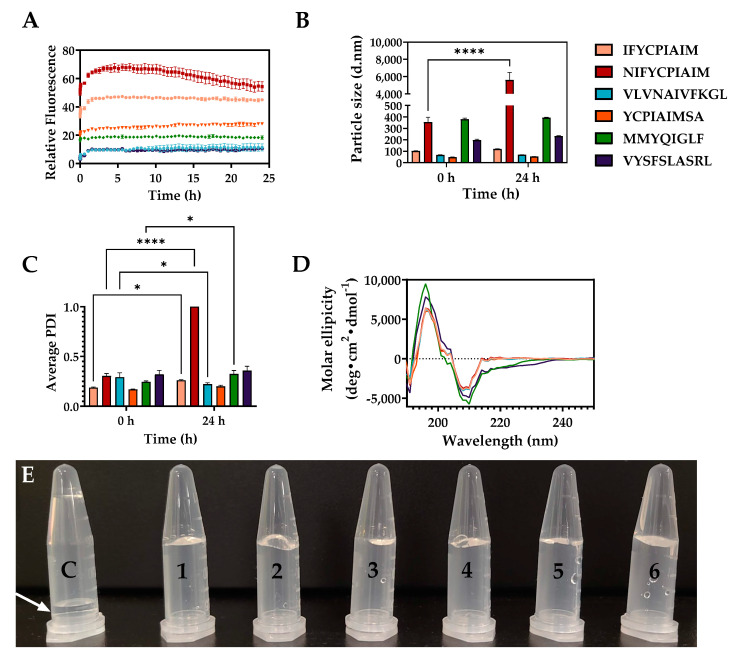
(**A**) Thioflavin T fluorescence kinetics, (**B**) particle diameter, (**C**) polydispersity index (PDI), and (**D**) circular dichroism spectra of the ovalbumin-derived peptides. (**E**) Formation of hydrogels that maintain their shape in test tubes. Control, “C“, indicates water mixed with the same concentration (*v*/*v*) of DMSO in water. * = significant (0.01 < *p* < 0.05), **** = extremely significant (*p* < 0.0001).

Peptide self-assembly was evaluated using ThT fluorescence kinetics. A rapid increase in fluorescence intensity was observed for all six peptides from t = 0 to 65 min (Figure 3A). This indicates that peptide self-assembly occurred spontaneously at room temperature following the addition of an anti-solvent. NIFYCPIAIM showed the highest increase in fluorescence intensity within the first 3–4 h, followed by a gradual decline in intensity for the subsequent 20 h. Additionally, dynamic light scattering analysis showed that NIFYCPIAIM formed the largest particle sizes of diameter up to 5611.3 nm and highest PDI of 1.0 (Figure 3B,C), indicating a heterogeneous mixture. The ThT fluorescence kinetics pattern and PDI of NIFYCPIAIM suggest fragmentation of the hydrogel possibly due to excessive water permeation into the gel on prolonged incubation, or the attainment of equilibrium between the monomer, oligomer, and fibrillary species of the peptide. The results suggest an ordered formation of the hydrogel rather than random aggregation, as fragmentation is a common initiator of secondary nucleation during fibrillation, even in the case of peptide hydrogelation [34]. The rapid aggregation of NIFYCPIAIM at the beginning was followed by an equilibrium state and a declining phase involving the fragmentation of existing fibrils. Following nucleation, self-assembly progresses rapidly until the free peptide concentration falls below the CAC; at this point, nanofibers remain in equilibrium with free peptides [35]. It is possible that NIFYCPIAIM self-assembly occurred rapidly to the point where an imbalance of the equilibrium resulted in a shift towards fragmentation of the formed fibrils towards the liberation of free peptides to re-establish equilibrium. This explains the decline in fluorescence intensity and high polydispersity index at 24 h. The increased fluorescence intensities for the other five peptides remained consistent up to 24 h, suggesting the stability of the hydrogels (Figure 3A). Similarly, these five peptides maintained consistent particle diameters, at t = 0 and 24 h, of intermediate sizes (393.7 and 232.5 nm for MMYQIGLF and VYSFSLASRL, respectively) and smaller sizes (120.1, 68.8, and 53.8 nm for IFYCPIAIM, VLVNAIVFKGL, and YCPIAIMSA, respectively) (Figure 3B). The particle diameters of MMYQIGLF and VYSFSLASRL were larger than those reported for Fmoc-FF/(FY)3 peptide nanogels (168 nm) [36], and all six ovalbumin peptides formed larger particles than the well-known nanofiber-forming RADA16 peptide hydrogel (10–50 nm) [37]. Apart from NIFYCPIAIM, the peptides also maintained homogeneous size distributions indicated by the lower polydispersity index values (PDI < 0.5), confirming the structural stability of the hydrogels. Circular dichroism was performed to elucidate the secondary structure of the peptides within the self-assembled structures (Figure 3D). Strong positive and negative peaks at 195 and 210 nm, respectively, are indicative of rich β-sheet structures [17,37] in all six peptides, with all peptides, except MMYQIGLF, having over 50% β-sheet content (Table 1). Similar β-sheet content (~50%) was reported for hydrogels from RADA16 peptide [37]. MMYQIGLF, on the other hand, had a lower β-sheet content of 31% (Figure 3D, Table 1), relative to the other five peptides. Despite the variations in fibrillar patterns over time, based on ThT fluorescence, the six ovalbumin-derived peptides formed physical hydrogels that maintained their shape on tube inversion, in contrast to the DMSO control (arrow), after a 24 h incubation (Figure 3E).

### 2.5. Microstructures of the Self-Assembling Ovalbumin Peptides

**Figure 4:**
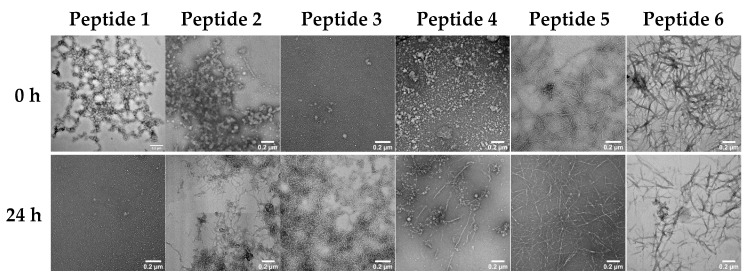
Transmission electron microscopy images of peptides at 0 h and after 24 h of incubation. Scale bars represent 0.2 µm. Peptide 1 = IFYCPIAIM; Peptide 2 = NIFYCPIAIM; Peptide 3 = VLVNAIVFKGL; Peptide 4 = YCPIAIMSA; Peptide 5 = MMYQIGLF; Peptide 6 = VYSFSLASRL.

TEM analysis showed varying amounts of intertwined fibrillary networks in all six peptides at 24 h, which are associated with hydrogel formation [7]. However, the peptides showed distinct morphologies at t = 0 and 24 h. For instance, IFYCPIAIM at t = 0 showed numerous amorphous aggregate clusters of spherical microstructures that developed into less dense long and thin fibrils at 24 h (Figure 4). NIFYCPIAIM had a similar amorphous aggregate morphology at t = 0, but it formed shorter, denser, and highly branched fibrils at 24 h (Figure 4). Amorphous aggregates or nanoclusters occur during peptide hydrogel formation and represent species typically found at the nucleation step of self-assembly [11]. YCPIAIMSA initially showed numerous amorphous aggregates that later developed into thin fibrils intercalated with amorphous aggregates (Figure 4). Notably, the peptides that have a common region, viz., IFYCPIAIM, NIFYCPIAIM and YCPIAIMSA, had similar behaviors, with their initial amorphous aggregates developing into variations of fibrillar structures. This highlights the shared sequence YCPIAIM as a structural feature of interest for elucidating the self-assembly and hydrogelation mechanisms and corroborates the sequence-dependent role of peptides in fibril formation [7,11,12]. In particular, the N-terminal IF and NIF of IFYCPIAIM, NIFYCPIAIM, respectively, resulted in an enhanced AGGRESCAN Na^4^vSS score, possibly because of more complex mechanisms involving these residues at play. Conversely, VLVNAIVFKGL formed a few scattered aggregates of undefined structural patterns at t = 0, which transformed into numerous short fibrils on prolonged incubation (Figure 4). This observation explains the small average particle diameter and low PDI observed for the peptide (Figure 3B,C). Microscale crystals formed by VLVNAIVFKGL after 48 h were similar in morphology to those formed by nucleo-tripeptide thymine-GFF and guanine-GFF hydrogels where π-π and hydrophobic interactions were thought to facilitate self-assembly [38]. With the presence of phenylalanine and other hydrophobic amino acids, glycine, alanine, valine, leucine, and isoleucine, it is possible that π-π and hydrophobic interactions facilitate the self-assembly of VLVNAIVFKGL resulting in the observed microscale crystals (Figure 4). Lastly, MMYQIGLF and VYSFSLASRL formed well-defined thin and thick ribbon-like fibrils, respectively at t = 0 (Figure 4). The self-assembled nanostructure of MMYQIGLF consisted of fibrils of 20.2 ± 7.1 nm in diameter with regular, repeated twists, similar to those of the SSP1 peptide [(VK)2-VDPPT-(KV)6-NH_2_] scaffold [39]. On the other hand, VYSFSLASRL nanofibrils appeared to have the cross β-sheet microstructure with a fibrillar diameter of 34.3 ± 14.3 nm, similar to the long, twisted but thicker microfibers of β-lactoglobulin [7]. The density of the fibrillar structures increased for MMYQIGLF but decreased for VYSFSLASRL at 24 h. It is likely that the nucleation step occurred rapidly for the latter resulting in the formation of denser fibrillar structures at the initial timepoint.

### 2.6. Mechanical Properties of the Peptide Hydrogels

**Figure 5:**
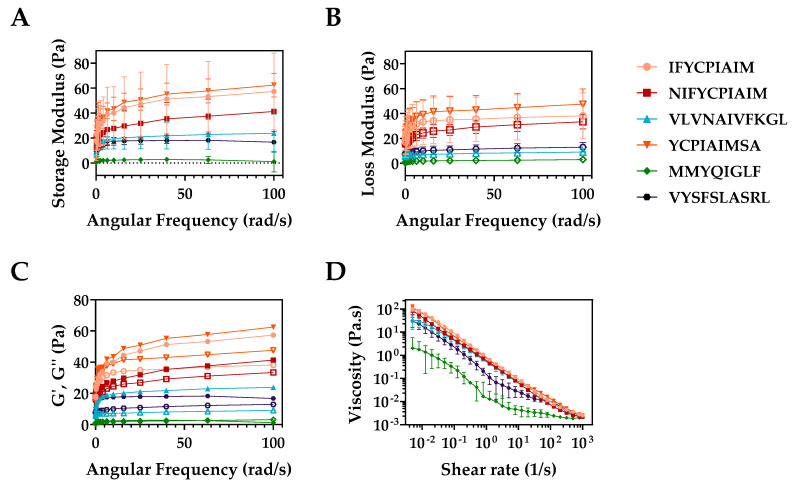
Biomechanical properties of ovalbumin-derived peptide self-assembly showing (**A**) the storage modulus (Gʹ), (**B**) loss modulus (Gʹʹ), and (**C**) both as a function of angular frequency, where Gʹ is the filled shapes and Gʹʹ is the corresponding open shapes (standard error bars removed for ease of viewing). (**D**) Flow sweep experiment of viscosity as a function of shear rate response of the self-assembling ovalbumin-derived peptides.

The biomechanical properties of hydrogels formed with the six ovalbumin-derived peptides were determined using rheological measurements to estimate the storage (G′) and loss moduli (G′′) (Figure 5A–C), and their flow properties (Figure 5D, Table 2). All six peptide hydrogels, as shown in Table 2, had viscoelastic pseudoplastic properties with a flow index n < 1. The order of viscosity was as follows: IFYCPIAIM > YCPIAIMSA > NIFYCPIAIM > VLVNAIVFKGL > VYSFSLASRL > MMYQIGLF, with the largest viscosity being observed with the sequence similar, IFYCPIAIM, YCPIAIMSA, and NIFYCPIAIM peptides (Figure 5D). This pattern confirms the importance of the shared region YCPIAIM, among the top-three peptides, in self-assembly and hydrogelation. Upon exerting shear stress, the peptide hydrogels lost their viscosity, demonstrating their non-Newtonian shear-thinning behavior (Figure 5D), but had the capacity to regain some level of viscosity upon release of the shear stress due to structural changes. This indicates the potential of these peptides for use as injectable gel-forming systems [40]. In these applications, gels must be able to exert shear-thinning properties when pressure is applied (e.g., injectable hydrogels) but retain their solid structure when stationary (thixotropic abilities). Hydrogels considered for injectable drug delivery systems are characterized by low storage and loss moduli as well as low viscosity [41]. All six ovalbumin peptide hydrogels had a storage modulus (G′) of 0–60 Pa (Figure 5A). Hydrogels with (G′) below 100 Pa are considered soft hydrogels, which are more suited for low elasticity applications, such as media for bone cell culture [11]. As shown in Figure 5C, all six peptide hydrogels had a storage modulus higher than the loss modulus (G′ > G′′), with IFYCPIAIM and YCPIAIM showing the largest difference. Despite the difference in values, the storage modulus does not dominate the loss modulus by at least one order of magnitude, suggesting that the resulting material of the self-assembled peptides are more elastic in nature than that of a hydrogel [42,43,44]. Peptide hydrogels with a loss factor tan δ < 1 have rheological properties similar to an elastic solid whereas hydrogels with tan δ > 1 behave like a viscous liquid [7]. Hydrogels with high tan δ values have weaker bonds between the networks [45], making them excellent for applications in wound dressing and tissue engineering as fillers in which weaker forming gels are more important [41]. Peptide hydrogelation is facilitated by covalent and non-covalent bonding. Covalent bonds, consisting of cross-linking via sulfhydryl bond formation, and non-covalent bonding, consisting of π-π interactions, hydrophobic interactions, van der Waals interactions, electrostatic interactions, and hydrogen bonding, are required to maintain the self-assembled three-dimensional structure [11,12], and are likely contributing to the lower loss factor observed with VLVNAIVFKGL and VYSFSLASRL. Consequently, hydrogels formed with VLVNAIVFKGL and VYSFSLASRL likely have stronger bonds holding their networks than the other hydrogels. Additional modification of the peptide structure or hydrogelation conditions could be explored to form tough hydrogels for use in biomedical applications such as wound dressing and tissue engineering.

## 3. Conclusions

In this study, we successfully identified ovalbumin peptides with self-assembling properties, while also exploring the effects of sequence structure on hydrogelation. Six ovalbumin peptides, IFYCPIAIM, NIFYCPIAIM, VLVNAIVFKGL, YCPIAIMSA, MMYQIGLF, and VYSFSLASRL, identified in pepsin-generated egg white protein hydrolysates by a combination of peptidomics and in silico analysis. Despite the abilities to form hydrogels, characterization of the peptides demonstrated a wide range of physicochemical and self-assembly properties. For example, NIFYCPIAIM showed the highest ThT fluorescence intensity, average particle diameter, and polydispersity index, indicating its high propensity for self-assembly. Interestingly, NIFYCPIAIM, IFYCPIAIM and YCPIAIMSA demonstrated similar fibrillation patterns characterized by the immediate formation of amorphous aggregates followed by reassembly into fibrillar networks after 24 h. The role of amino acid sequence on the hydrogelation properties is evident in these three peptides as they share the common sequence YCPIAIM. Conversely, MMYQIGLF and VYSFSLASRL demonstrated the immediate formation of interconnected networks of twisted thin and thick ribbon fibrils, respectively, corresponding to their particle characteristics, despite having lower ThT fluorescence intensities than the other peptides. Despite the characteristic differences, rheological measurements showed that all six ovalbumin peptides formed soft gels, indicated by the low Gʹ, of viscous liquid nature (Gʹ > Gʹʹ). As hydrogelation is largely influenced by environmental factors such as solvent composition, temperature, pH, and ionic strength, future studies are needed to optimize these conditions in order to enhance the biomechanical properties and create more robust hydrogels for use in biomaterial, biosensing, and food applications. Taken together, the findings demonstrate that the six ovalbumin-derived self-assembling peptides form hydrogels with promising biomechanical properties for biomaterial applications such as injectable gel-forming and drug delivery systems.

## 4. Materials and Methods

### 4.1. Materials and Chemicals

Egg white (10 g protein/100 g egg white) was purchased from a local store in Ottawa (ON, Canada). 8-Anilino-1-naphthalenenesulfonic acid (ANS), ophthaldialdehyde (OPA), sodium dodecyl sulfate, dithiothreitol, L-serine, glycine, urea, 5,5’-dithiobis-[2-nitrobenzoic acid], pepsin from porcine gastric mucosa, thioflavin T (ThT), and DMSO were purchased from MilliporeSigma (Oakville, ON, Canada). UranyLess counterstain was purchased from Electron Microscopy Sciences (Hatfield, PA, USA).

### 4.2. Preparation of Egg White Protein (EWP) Hydrolysate and Aggregate Samples

The egg white solution was diluted with deionized water to 0.1% (*w*/*v*, protein basis) and hydrolyzed for 5 h with pepsin at pH 2.0 (adjusted with 1 M HCl, 37 ℃) and an enzyme/substrate ratio of 1:100 (*w*/*w*, protein basis). After hydrolysis, pepsin was deactivated by heating at 95 ℃ for 15 min and the mixture was adjusted to pH 7.0 using 1 M NaOH. The supernatant was collected after centrifugation at 10,000× *g* for 10 min, freeze-dried, and used as the EWP hydrolysates [26]. To prepare the aggregate sample, EWP hydrolysate powder was suspended in deionized water (40%, *w*/*v*), incubated with shaking (40 rpm) at 37 ℃ for 16 h, and freeze dried to collect the powder sample for further analysis.

### 4.3. Characterization of EWP Hydrolysate and Aggregate Samples

Free amino nitrogen of the EWP hydrolysate and aggregate samples was determined using the OPA method [46]. Serine was used as the standard and the free amino nitrogen was calculated as milliequiv of serine NH_2_/g sample [26]. Particle size and polydispersity index (PDI) of samples at a concentration of 1 mg/mL were analyzed by dynamic light scattering using Zetasizer Nano ZS (Malvern Instruments Ltd., Worcestershire, UK) at 25 °C [26]; dynamic light scattering effect was analyzed in Milli-Q water (pH 7, refractive index 1.330, viscosity 0.8872 cP and dielectric constant 78.5) with Smoluchowski model at F(ka) 1.50 and backscattered angle of 173°. To evaluate peptide aggregation and gel formation, the free sulfhydryl content was determined according to the method reported by Hong et al. [47], and surface hydrophobicity (S0) was determined using ANS as the fluorescent probe [48].

ThT (10 µM) fluorescence assay was conducted at the sample concentrations of 1.6, 1.0, 0.5, 0.25, and 0.125 mg/mL in black 96-well microplates with clear bottoms, at a final volume of 200 µL. Samples were prepared in triplicates and fluorescence intensity measurements were recorded from the bottom of the plate at λ_ex_ 430 nm and λ_em_ 480 nm using the Spark multimode microplate reader (Tecan, Stockholm, Sweden). The aggregates were imaged at 1.6 mg/mL by mounting 5 µL of sample on microscope slides and securing with a coverslip prior to imaging. Images were obtained using the Axio Imager 2 fluorescence microscope equipped with an Axiocam 506 camera (Carl Zeiss, Germany) using the fluorescein isothiocyanate channel (λ_ex_ 495 nm and λ_em_ 519 nm). The images were processed using the Zen 2.3 pro software (Carl Zeiss, Germany).

### 4.4. LC-MS/MS Analysis

EWP hydrolysate (~5 µg of protein) were analyzed by nLC-MS/MS and the data was processed as previously described [49]. In brief, mass spectra were generated on an Orbitrap Fusin mass spectrometer (Thermo Fisher Scientific, Waltham, MA, USA) coupled to an UltiMate 3000 nanoRSLC (Dionex, Thermo Fisher Scientific, Waltham, MA, USA).

Spectral data was analyzed with MaxQuant (version 2.0.3.1) and the Andromeda search engine [50,51]. Peptides were searched against a Gallus gallus fasta file obtained from UniProt and common contaminant protein sequences. Peptides ranging from 6 to 25 amino acid residues were searched with unspecific cleavage. N-terminal acetylation and methionine oxidation were considered variable modifications, and cysteine carbamidomethylation as a fixed modification. An initial precursor mass deviation of up to 10 ppm and a fragment mass deviation of 0.5 were used. A false discovery rate (FDR) 0.01 was applied to both peptide and protein identifications, with a reverse sequence decoy database.

Proteins and peptides identified from the reverse database were removed. The MaxQuant output tables were then loaded in the R programming environment. Peptide properties were obtained from the Peptides R package [31].

### 4.5. Prediction of Aggregation Propensity of Ovalbumin-derived Peptides Using AGGRESCAN

Ovalbumin-derived peptides from the aggregate sample were selected and analyzed using AGGRESCAN, a web-based tool used for the prediction of aggregation propensity of peptides. Regions of five or more consecutive residues in a sequence without proline is termed an aggregation “Hot Spot”. The AGGRESCAN algorithm predicts the tendency of each residue within a sequence to aggregate and compares all sequences within the same data set [29,52].

### 4.6. Peptide Synthesis

Six aggregation-prone ovalbumin peptides were synthesized and supplied as white powders by Bootech BioScience and Technology Co., Ltd. (Shanghai, China). Purity was determined by the supplier to be >95% after observing the peptide peak by analytical high-performance liquid chromatography and mass spectrometry analysis.

### 4.7. Peptide Hydrogel Formation

Stock solutions of 20 mM peptide were prepared in DMSO. Then, at a final concentration of 40 µM in 500 µL, peptides were combined with deionized water and vortexed briefly to initiate hydrogel formation. Samples were left at room temperature (21 °C) under static conditions for 24 h for hydrogel formation. The total concentration of DMSO was kept to 0.2–2% (*v*/*v*) to prevent interference with hydrogelation [53]. For physical observation of hydrogels, peptides were prepared as described and a sample of the equivalent concentration of DMSO in water was prepared as the control.

### 4.8. Thioflavin T Assays

ThT fluorescence assay was used to determine the CAC of the peptides. An aqueous solution of the peptides (0.001–100 µM) was mixed with 10 µM ThT in a black 96-well microplate with a clear bottom. Fluorescence intensity was recorded from the bottom of the plate at λ_ex_ 430 nm and λ_em_ 480 nm using the Spark multimode microplate reader.

ThT kinetic fluorescence assays was used to monitor self-assembly of the six ovalbumin-derived peptides. The peptides (40 µM) and ThT (10 µM) were mixed in a black 96-well microplate with a clear bottom. Samples were shaken for 10 s at 1440 rpm and fluorescence intensity was recorded in kinetic mode from the bottom of the plate at λ_ex_ 430 nm and λ_em_ 480 nm every 30 s for the first 5 min and then every 30 min for 24 h using the Spark multimode microplate reader. Baseline subtraction was done against the fluorescence of ThT in buffer.

### 4.9. Dynamic Light Scattering

The average particle diameter and polydispersity index of the peptides were recorded before (0 h) and after 24 h of incubation at room temperature as previously described. Aqueous solution of 40 µM peptide was used for the analysis at 21 °C.

### 4.10. Circular Dichroism Spectroscopy

Circular dichroism was performed to determine the secondary structure of the self-assembled peptides. A 400 mg/mL stock of each peptide was prepared in DMSO and diluted to a final concentration of 0.4 mg/mL in deionized water (final concentration of DMSO was 0.1% (*v*/*v*)), and then incubated at room temperature (21 °C), undisturbed, for 24 h. Spectroscopic measurements were completed using the Jasco J-715 Circular Dichroism spectrophotometer (Jasco Corp., Tokyo, Japan), with a quartz cuvette (path length = 1 mm) at room temperature under nitrogen gas. Three scans were recorded at a wavelength range of 190–250 nm and scanning speed of 100 nm/sec and then averaged. Baseline subtraction was done using a deionized water and DMSO (0.1% *v*/*v*) blank and the results were converted to mean residue ellipticity (deg × cm^2^ × dmol^−1^) using a mean residue weight of 110.74 Da and peptide concentration of 0.4 mg/mL on the CDToolX software [54]. Plotting and smoothing of the resulting spectra were performed using GraphPad Prism version 9.2.0 for Windows (GraphPad Software, La Jolla, CA, USA). Secondary structure approximation was achieved using the CD fitting software, BeStSel [32], using the wavelength range, 190–250 nm.

### 4.11. Transmission Electron Microscopy

A 10-µL aliquot of peptide (40 µM in water), at 0 h and 24 h of incubation at room temperature, was placed on 300-mesh Formvar-carbon coated copper grid and incubated for 5 min for absorption. Excess sample was blotted with a Kimwipe, and the grids were counterstained with UranyLess of 1 min in the dark. Excess stain was blotted again, and the loaded grids were imaged using the JEM-1400 Flash Electron Microscope (JEOL, Tokyo, Japan) at an accelerating voltage of 120 kV. The images were processed and quantified using ImageJ 1.53c software (NIH, Bethesda, MD, USA) [55].

### 4.12. Rheological Measurements

Rheological properties of the peptides were determined using the Discovery Hybrid Rheometer HR-2 (TA Instruments Ltd., New Castle, USA) containing a cone (2°, 40 mm diameter, 50 µm truncation gap) and a Peltier plate steel measuring system with a gap height set to 1 mm. The peptide hydrogel sample (1.5 mL of 40 µM in distilled water) was gently loaded onto the lower testing plate and equilibrated for 5 s at 25  °C. A flow sweep test was performed by setting the shear rate from 5 × 10^−2^ s^−1^ to 1000 s^−1^ to study the flow behavior. An oscillation frequency scan was run from 0.1 to 100 rad/s using a fixed strain of 1% to record the storage modulus (G′), loss modulus (G″), and loss tangent (tan δ).

### 4.13. Statistical Analysis

Statistical analysis was performed using a one-way or two-way analysis of variance with GraphPad Prism version 9.2.0 for Windows (GraphPad Software, La Jolla, CA, USA). Significant difference between the mean values was defined as *p* < 0.05 using the unpaired t-test or Šídák’s multiple comparisons test.

## Figures and Tables

**Table 1:** Physicochemical properties and aggregation propensity of the ovalbumin-derived peptides.

Sequence	Location in ovalbumin	Experimental MW (Da)	Na^4^vSS	Hydrophobicity score	β-sheet %
IFYCPIAIM	28–36	1069.53	79.1	2.18	54.3
NIFYCPIAIM	27–36	1183.58	71.6	1.61	53.6
VLVNAIVFKGL	174–184	1171.73	54.2	1.95	52.7
YCPIAIMSA	30–38	967.451	52.8	1.48	51.0
MMYQIGLF	211–218	1001.47	46.0	1.21	31.0
VYSFSLASRL	97–106	1141.61	32.0	0.82	52.0

Abbreviations: MW, molecular weight; Na^4^vSS, normalized a^4^vSS for 100 residues, where a4v represents an experimentally derived parameter that represents the aggregation profile [29]. Na^4^vSS estimates the average aggregation propensity of a peptide sequence. Hydrophobicity score based on the Kyte-Doolittle scale [30], calculated using the *Peptides* R package [31]. The β-sheet percentage was derived from circular dichroism and calculated using BeStSel [32].

**Table 2:** Flow behavior of the ovalbumin-derived peptide hydrogels.

Sequence	Flow index (n) × 10^−3^	Consistency (K)	G′/G′′ (tan δ)
IFYCPIAIM	1.23 ± 0.06 ^c^	0.824 ± 0.045 ^a^	0.928 ± 0.165 ^a^
NIFYCPIAIM	1.53 ± 0.06 ^c^	0.542 ± 0.036 ^c^	0.978 ± 0.050 ^a^
VLVNAIVFKGL	1.77 ± 0.38 ^c^	0.506 ± 0.121 ^c^	0.521 ± 0.162 ^a^
YCPIAIMSA	1.40 ± 0.10 ^c^	0.795 ± 0.050 ^b^	1.106 ± 0.431 ^a^
MMYQIGLF	5.35 ± 0.07 ^a^	0.032 ± 0.002 ^e^	1.182 ± 1.152 ^a^
VYSFSLASRL	3.10 ± 0.53 ^b^	0.245 ± 0.095 ^d^	0.657 ± 0.138 ^a^

Superscripts with different letters in each column denotes statistically different (*p* < 0.05) mean values.

## Data Availability

All the data in this study are included within the article and Supplementary Materials.

## Supplementary Materials

The following supporting information can be downloaded at: https://www.mdpi.com/article/10.3390/gels8100641/s1, Table S1: Aggregation propensity of all ovalbumin-derived peptides as predicted using AGGRESCAN analysis. Figure S1: Critical aggregation concentration (CAC) of the six ovalbumin-derived peptides.

## References

[1] Chang C., Lahti T., Tanaka T., Nickerson M.T. (2018). Egg proteins: Fractionation, bioactive peptides and allergenicity. J. Sci. Food Agric..

[2] Bhat Z.F., Morton J.D., Bekhit A.E.D.A., Kumar S., Bhat H.F. (2021). Effect of processing technologies on the digestibility of egg proteins. Compr. Rev. Food Sci. Food Saf..

[3] Ma X., Liang R., Xing Q., Lozano-Ojalvo D. (2020). Can food processing produce hypoallergenic egg?. J. Food Sci..

[4] Mahdavi S., Amirsadeghi A., Jafari A., Niknezhad S.V., Bencherif S.A. (2021). Avian egg: A multifaceted biomaterial for tissue engineering. Ind. Eng. Chem. Res..

[5] Farjami T., Babaei J., Nau F., Dupont D., Madadlou A. (2021). Effects of thermal, non-thermal and emulsification processes on the gastrointestinal digestibility of egg white proteins. Trends Food Sci. Technol..

[6] Lv X., Huang X., Ma B., Chen Y., Batool Z., Fu X., Jin Y. (2022). Modification methods and applications of egg protein gel properties: A review. Compr. Rev. Food Sci. Food Saf..

[7] Pugliese R., Gelain F., Arnoldi A., Lammi C., Udenigwe C.C. (2021). Chapter 6: Chemistry and functional roles of food protein hydrogels. Food Proteins and Peptides: Emerging Biofunctions, Food and Biomaterial Applications.

[8] Rathnapala E.C.N., Ahn D.U., Abeyrathne S. (2021). Functional properties of ovotransferrin from chicken egg white and its derived peptides: A review. Food Sci. Biotechnol..

[9] Benedé S., Molina E. (2020). Chicken egg proteins and derived peptides with antioxidant properties. Foods.

[10] Mildenberger J., Remm M., Atanassova M. (2021). Self-assembly potential of bioactive peptides from norwegian sea cucumber parastichopus tremulus for development of functional hydrogels. LWT.

[11] de Leon Rodriguez L.M., Hemar Y. (2020). Prospecting the applications and discovery of peptide hydrogels in food. Trends Food Sci. Technol..

[12] Mondal S., Das S., Nandi A.K. (2020). A review on recent advances in polymer and peptide hydrogels. Soft Matter.

[13] Raza F., Zhu Y., Chen L., You X., Zhang J., Khan A., Khan M.W., Hasnat M., Zafar H., Wu J. (2019). Paclitaxel-loaded pH responsive hydrogel based on self-assembled peptides for tumor targeting. Biomater. Sci..

[14] Lammi C., Bollati C., Gelain F., Arnoldi A., Pugliese R. (2019). Enhancement of the stability and anti-DPPIV activity of hempseed hydrolysates through self-assembling peptide-based hydrogels. Front. Chem..

[15] Pramanik B., Ahmed S. (2022). Peptide-based low molecular weight photosensitive supramolecular gelators. Gels.

[16] Nandi N., Gayen K., Ghosh S., Bhunia D., Kirkham S., Sen S.K., Ghosh S., Hamley I.W., Banerjee A. (2017). Amphiphilic peptide-based supramolecular, noncytotoxic, stimuli-responsive hydrogels with antibacterial activity. Biomacromolecules.

[17] Pugliese R., Fontana F., Marchini A., Gelain F. (2018). Branched peptides integrate into self-assembled nanostructures and enhance biomechanics of peptidic hydrogels. Acta Biomater..

[18] Omana D.A., Liang Y., Kav N.N.V., Wu J. (2011). Proteomic analysis of egg white proteins during storage. Proteomics.

[19] Wan M., Huang Z., Yang X., Chen Q., Chen L., Liang S., Zeng Q., Zhang R., Dong L., Su D. (2022). Fabrication and interaction mechanism of ovalbumin-based nanocarriers for metallic ion encapsulation. Int. J. Food Sci. Technol..

[20] Hassan N., Messina P.V., Dodero V.I., Ruso J.M. (2011). Rheological properties of ovalbumin hydrogels as affected by surfactants addition. Int. J. Biol. Macromol..

[21] Yu S., Hu J., Pan X., Yao P., Jiang M. (2006). Stable and pH-sensitive nanogels prepared by self-assembly of chitosan and ovalbumin. Langmuir.

[22] Kamalov M., Kählig H., Rentenberger C., Müllner A.R.M., Peterlik H., Becker C.F.W. (2019). Ovalbumin epitope SIINFEKL self-assembles into a supramolecular hydrogel. Sci. Rep..

[23] Malmos K.G., Blancas-Mejia L.M., Weber B., Buchner J., Ramirez-Alvarado M., Naiki H., Otzen D. (2017). ThT 101: A primer on the use of thioflavin t to investigate amyloid formation. Amyloid.

[24] Wang Q., Jiang J., Xiong Y.L. (2019). Genipin-aided protein cross-linking to modify structural and rheological properties of emulsion-filled hempseed protein hydrogels. J. Agric. Food Chem..

[25] Pugliese R., Bartolomei M., Bollati C., Boschin G., Arnoldi A., Lammi C. (2022). Gel-forming of self-assembling peptides functionalized with food bioactive motifs modulate DPP-IV and ACE inhibitory activity in human intestinal caco-2 cells. Biomedicines.

[26] Sun X., Acquah C., Gazme B., Boachie R.T., Nwachukwu I.D., Udenigwe C.C. (2021). Mechanisms of plastein formation influence the IGE-binding activity of egg white protein hydrolysates after simulated static digestion. Food Chem..

[27] Shen W., Lammertink R.G.H., Sakata J.K., Kornfield J.A., Tirrell D.A. (2005). Assembly of an artificial protein hydrogel through leucine zipper aggregation and bisulfide bond formation. Macromolecules.

[28] Mirzaei M., Mirdamadi S., Safavi M., Soleymanzadeh N. (2020). The stability of antioxidant and ACE-inhibitory peptides as influenced by peptide sequences. LWT.

[29] Conchillo-Solé O., de Groot N.S., Avilés F.X., Vendrell J., Daura X., Ventura S. (2007). AGGRESCAN: A server for the prediction and evaluation of “hot spots” of aggregation in polypeptides. BMC Bioinform..

[30] Kyte J., Doolittle R.F. (1982). A simple method for displaying the hydropathic character of a protein. J. Mol. Biol..

[31] Osorio D., Rondon-Villarreal P., Torres R. (2015). Peptides: A package for data mining of antimicrobial peptides. The R Journal.

[32] Micsonai A., Wien F., Bulyáki É., Kun J., Moussong É., Lee Y.H., Goto Y., Réfrégiers M., Kardos J. (2018). BeStSel: A web server for accurate protein secondary structure prediction and fold recognition from the circular dichroism spectra. Nucleic Acids Res..

[33] LeVine H. (1999). Quantification of β-sheet amyloid fibril structures with thioflavin t. Methods Enzymol..

[34] Braun G.A., Ary B.E., Dear A.J., Rohn M.C.H., Payson A.M., Lee D.S.M., Parry R.C., Friedman C., Knowles T.P.J., Linse S. (2020). On the mechanism of self-assembly by a hydrogel-forming peptide. Biomacromolecules.

[35] Yuan C., Levin A., Chen W., Xing R., Zou Q., Herling T.W., Challa P.K., Knowles T.P.J., Yan X. (2019). Nucleation and growth of amino acid and peptide supramolecular polymers through liquid–liquid phase separation. Angew. Chem..

[36] Gallo E., Diaferia C., Rosa E., Smaldone G., Morelli G., Accardo A. (2021). Peptide-based hydrogels and nanogels for delivery of doxorubicin. Int. J. Nanomed..

[37] Liu J., Zhang L., Yang Z., Zhao X. (2011). Controlled release of paclitaxel from a self-assembling peptide hydrogel formed in situ and antitumor study in vitro. Int. J. Nanomed..

[38] Baek K., Noblett A.D., Ren P., Suggs L.J. (2019). Design and characterization of nucleopeptides for hydrogel self-assembly. ACS Appl Bio. Mater..

[39] Hule R.A., Nagarkar R.P., Hammouda B., Schneider J.P., Pochan D.J. (2009). Dependence of self-assembled peptide hydrogel network structure on local fibril nanostructure. Macromolecules.

[40] Yan C., Pochan D.J. (2010). Rheological properties of peptide-based hydrogels for biomedical and other applications. Chem. Soc. Rev..

[41] Stojkov G., Niyazov Z., Picchioni F., Bose R.K. (2021). Relationship between structure and rheology of hydrogels for various applications. Gels.

[42] Adams D.J. (2022). Personal perspective on understanding low molecular weight gels. J. Am. Chem. Soc..

[43] Sathaye S., Mbi A., Sonmez C., Chen Y., Blair D.L., Schneider J.P., Pochan D.J. (2015). Rheology of peptide- and protein-based physical hydrogels: Are everyday measurements just scratching the surface?. Wiley Interdiscip. Rev. Nanomed. Nanobiotechnol..

[44] Draper E.R., Su H., Brasnett C., Poole R.J., Rogers S., Cui H., Seddon A., Adams D.J. (2017). Opening a can of worm(-like micelle)s: The effect of temperature of solutions of functionalized dipeptides. Angew. Chem. Int. Ed..

[45] Zhang J., Wang G., Liang Q., Cai W., Zhang Q. (2019). Rheological and microstructural properties of gelatin b/tara gum hydrogels: Effect of protein/polysaccharide ratio, pH and salt addition. LWT.

[46] Nielsen P.M., Petersen D., Dambmann C. (2001). Improved method for determining food protein degree of hydrolysis. J. Food Sci..

[47] Hong G.P., Avramenko N., Stone A., Abbott D., Classen H., Nickerson M. (2012). Extractability and molecular modifications of gliadin and glutenin proteins withdrawn from different stages of a commercial ethanol fuel/distillers dried grains with solubles process using a wheat feedstock. Cereal Chem..

[48] Hayakawa S., Nakai S. (1985). Relationships of hydrophobicity and net charge to the solubility of milk and soy proteins. J. Food Sci..

[49] Boachie R.T., Okagu O.D., Abioye R., Hüttmann N., Oliviero T., Capuano E., Fogliano V., Udenigwe C.C. (2022). Lentil protein and tannic acid interaction limits in vitro peptic hydrolysis and alters peptidomic profiles of the proteins. J. Agric. Food Chem..

[50] Cox J., Mann M. (2008). MaxQuant enables high peptide identification rates, individualized p.p.b.-range mass accuracies and proteome-wide protein quantification. Nat. Biotechnol..

[51] Cox J., Neuhauser N., Michalski A., Scheltema R.A., Olsen J.V., Mann M. (2011). Andromeda: A peptide search engine integrated into the MaxQuant environment. J. Proteome Res..

[52] de Groot N.S., Castillo V., Graña-Montes R., Ventura S. (2012). AGGRESCAN: Method, application, and perspectives for drug design. Methods Mol. Biol..

[53] Tjernberg A., Markova N., Griffiths W.J., Hallén D. (2006). DMSO-related effects in protein characterization. SLAS Discov..

[54] Miles A.J., Wallace B.A. (2018). CDtoolX, a downloadable software package for processing and analyses of circular dichroism spectroscopic data. Protein Sci..

[55] Schindelin J., Arganda-Carreras I., Frise E., Kaynig V., Longair M., Pietzsch T., Preibisch S., Rueden C., Saalfeld S., Schmid B. (2012). Fiji: An open-source platform for biological-image analysis. Nat. Methods.

