# Supplementary material for: Self-assembly and Hydrogelation Properties of Peptides Derived from Peptic Cleavage of Aggregation-prone Regions of Ovalbumin
Source: Gels. 2022 Oct 9;8(10):641. doi: 10.3390/gels8100641 (PMC9601990; doi:10.3390/gels8100641)
Supplement: Supplementary file 1 [file gels-08-00641-s001.zip › Figure S1_CAC determination.pdf]

Self-assembly and Hydrogelation Properties of Peptides Derived from Peptic Cleavage of Aggregation-prone Regions of Ovalbumin

Raliat O. Abioye [1], Caleb Acquah [2], Pei Chun Queenie Hsu [1,3], Nico Hüttmann [1], Xiaohong Sun [2,4] and Chibuike C. Udenigwe [1,2,*]

[1] Department of Chemistry and Biomolecular Sciences, Faculty of Science, University of Ottawa, Ottawa, Ontario, K1N 6N5, Canada
[2] School of Nutrition Sciences, Faculty of Health Sciences, University of Ottawa, Ottawa, Ontario, K1H 8M5, Canada
[3] Bioengineering, Faculty of Life Science, Rhine-Wall University of Applied Sciences, Marie-Curie-Straße 1, 47533 Kleve, Germany
[4] Department of Plant, Food & Environmental Sciences, Dalhousie University, Truro, NS, B2N 5E3, Canada
* Correspondence: cudenigw@uottawa.ca; Tel.: +1-(613)-562-5800 (ext. 6539).

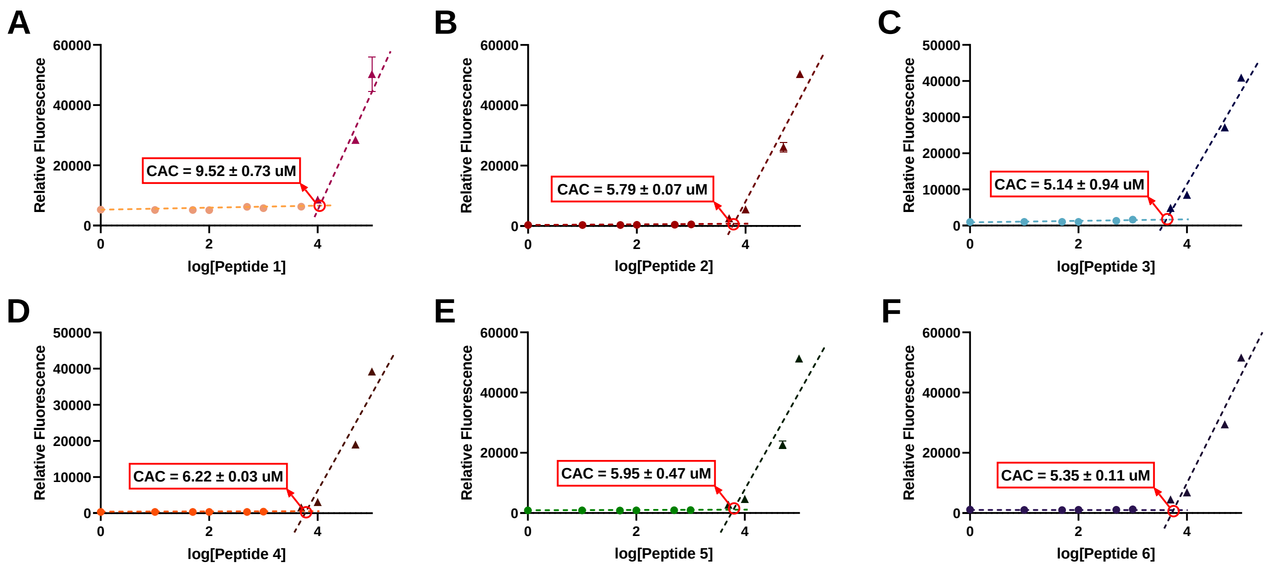

Figure S1. Critical aggregation concentration (CAC) of the six ovalbumin-derived peptides. **(A)** Peptide 1 = IFYCPIAIM, **(B)** Peptide 2 = NIFYCPIAIM, **(C)** Peptide 3 = VLVNAIVFKGL, **(D)** Peptide 4 = YCPIAIMSA, **(E)** Peptide 5 = MMYQIGLF, and **(F)** Peptide 6 = VYSFSLASRL.
